# Thermoregulatory costs in molting Antarctic Weddell seals: impacts of physiological and environmental conditions

**DOI:** 10.1093/conphys/coaa022

**Published:** 2020-04-04

**Authors:** Skyla M Walcott, Amy L Kirkham, Jennifer M Burns

**Affiliations:** 1 Department of Biological Sciences, University of Alaska Anchorage, 3101 Science Circle, Anchorage, AK 99508, USA; 2 College of Fisheries and Ocean Sciences, University of Alaska Fairbanks, 17101 Point Lena Loop Road Juneau, AK 99801, USA

**Keywords:** Heat flux, pinniped, surface temperature, thermoregulation, weather conditions

## Abstract

For polar marine mammals, the energetic cost of thermoregulation depends on ambient conditions in the highly variable surrounding environment. Heat conservation strategies used by pinnipeds to reduce total heat loss include small surface area to volume ratios, the ability to limit perfusion and thick subcutaneous blubber layers. There are limits to how cool the skin surface may remain without compromising function, especially during the annual pelage molt, when hair and skin are replaced. To determine if actively molting seals incur higher thermoregulatory costs, surface temperature (ST) and heat flux (HF) were measured in 93 adult female Weddell seals (*Leptonychotes weddellii*) both prior to and during the active molting period using direct sensors and infrared imaging. Linear mixed-effect models revealed that ST increased significantly with increased ambient temperature and decreased wind speed (contributing 44.6 and 41.7% of the attributed variance, respectively). Seal STs were not impacted by molt status, but were maintained at 11.2 ± 0.3°C warmer than the ambient temperature. Infrared imaging results averaged 15.1 ± 1.4°C warmer than direct ST measurements. In contrast, HF was significantly higher in seals in early molting stages compared to the pre-molt season ( *P* < 0.001) and molt status accounted for 66.5% of the variance in HF. Thermoregulatory costs calculated from estimated basal metabolic rate and measured HF were more than double for molting seals as compared to those in pre-molt. This suggests that perfusion is increased during molt to support follicle development, despite the increased energetic costs associated with higher HF rates. Because ST, HF and thermoregulatory costs are strongly influenced by ambient conditions, molt timing is likely under selective pressure to occur during the warmest period of the year. Shifts in environmental conditions that delay molt phenology or increase HF rates could negatively impact seal populations by further increasing thermoregulatory costs.

## Introduction

Antarctic marine mammals face complex thermoregulatory challenges, as they must conserve heat in both air and water at ambient temperatures that are well below their core body temperature (37°C). Heat loss is primarily driven by the temperature difference (∆*T*) between the animal’s surface and the outside environment [∆*T* = *T*_surface_ − *T*_environment_ (Kvadsheim and Folkow, 1997; Bejan and Kraus, 2003)], and it can vary substantially in response to changes in physiological and ambient conditions (Norris *et al.*, 2010; Mellish *et al.*, 2015), as well as differences in haul-out substrates (conduction: land or ice) or mediums (radiation, convection: air or water). To reduce the rate at which metabolic heat is lost to the environment, marine mammals have a suite of adaptations to reduce ∆*T* when in both air and water. First, fur insulates the animal from the surrounding environment by trapping air close to the skin. Insulation capacity increases with the length and density of the pelage (Frisch *et al.*, 1974; Kvadsheim and Aarseth, 2002). Many marine mammal species also have a thick layer of subcutaneous blubber that provides substantial insulation (Scholander *et al.*, 1950; Worthy, 1991; Kvadsheim *et al.*, 1994). Supplementary adaptations that reduce the temperature gradient and therefore heat loss include the ability to precisely regulate blood flow from the warm core to the peripheral tissues with arteriovenous anastomoses (Molyneux and Bryden, 1978; Krmpotic *et al.*, 2018). Limiting perfusion to the surface of the skin allows it to be maintained at a temperature closer to that of the environment, reducing the gradient and conserving heat. Alternatively, perfusion can be increased in specific regions (usually ‘hot spots’ along the trunk) to dump heat when needed (Rodríguez-Prieto *et al.*, 2013; Williams *et al.*, 1999; Liwang, 2008; Mellish *et al.*, 2015). The effectiveness of these adaptations at controlling heat loss is reflected by the fact that resting metabolic rates for marine mammals are similar to those of terrestrial mammals, even in species that inhabit cold polar regions and swim in ice-covered waters (Scholander *et al.*, 1950; Williams *et al.*, 1999; Mellish *et al.*, 2015).

Most aquatic mammals undergo an annual molt (Beltran *et al*., 2018). In pinnipeds, the molt occurs when old and worn fur [and in some cases the epidermal layer (Ling, 1972)] is replaced over a period of ~1 month (Ashwell-Erickson *et al.*, 1986; Hindell and Burton, 1988; Boyd *et al.*, 1993). During the molt, hair follicles likely require more constant perfusion than during non-molting periods (Paterson *et al.*, 2012). This requirement may preclude the use of vasoconstriction to limit heat loss, leading to increased thermoregulatory costs and therefore increased metabolic rates in some species (Hansen *et al.*, 1995; Ladds *et al.*, 2017). To offset these additional thermoregulatory costs, fully aquatic species may move to warmer waters for the molt (Boily, 1995), while many semi-aquatic pinnipeds alter their behaviour and spend more time hauled-out to reduce high heat loss to cold waters (Calambokidis *et al.*, 1987; Carlens *et al.*, 2006). However, hauling-out more frequently reduces pinnipeds’ access to underwater prey resources, which can lead to catabolism of blubber energy reserves (Ryg *et al.*, 1988; Noren *et al.*, 2013). Thinning of the blubber layer reduces its insulative capacity (Parry, 1949; Iverson, 2009), potentially increasing thermoregulatory costs.

While several physiological properties [mass (M), surface area (SA), blubber thickness (B) and molt status (MS)] can impact the thermoregulatory costs of molt (Kleiber, 1947; Pearson *et al.*, 2014; Ladds *et al.*, 2017), thermoregulatory costs are also influenced by environmental conditions such as ambient temperature (AT), wind speed (WS), relative humidity (RH) and solar radiation [SR, (Williams *et al.*, 2011; Chaise *et al.*, 2018; Erdsack *et al.*, 2018)]. Managing the energetic cost of molt may be particularly important for Antarctic pinnipeds, because there is only a brief period of high primary productivity (Smith *et al.*, 1996) and warmer temperatures (Zhang *et al.*, 2018) during summer. While summer offers more abundant foraging opportunities than the rest of the year (Smith and Nelson, 1986; Smith *et al.*, 1991; Schofield *et al.*, 2010), it is also the time when seals must spend more time hauled-out to nurse offspring and molt. Therefore, the short summer season likely constrains the timing of both reproduction and molt. As a result, in Weddell seals (*Leptonychotes weddellii*), the time between these two critical life history events is only ~ 7 weeks (Lugg, 1966; Castellini *et al.*, 2009), which is much shorter than that of more temperate species (Calambokidis *et al.*, 1987; Lewis *et al.*, 2004). For Weddell seals, condensing the period between the end of the breeding season and the start of the pelage molt gives reproductive females less time to regain mass and energy reserves delivered to pups. Therefore, unless they delay the molt until when weather conditions are poorer, they may start molting with thinner blubber layers. Because even warm ambient summer conditions are well below Weddell seal core body temperatures, reduced insulation likely leads to increased thermoregulatory costs during the molt, particularly if skin is maintained warmer and/or is more perfused as compared to non-molting periods.

To determine the thermoregulatory cost of the annual pelage molt in Weddell seals, we (i) determined surface temperatures (ST) and heat flux (HF) rates using direct thermocouple and HF measurements, (ii) identified the intrinsic and extrinsic factors that influenced ST and HF, (iii) used thermal infrared imaging (IRT) to determine regional patterns of perfusion during the molt and (iv) calculated the heat flux from obligatory thermoregulation (HFTR) and compared HF costs between pre-molt and actively molting seals. By determining the total amount of heat loss by molting Weddell seals, this study provides the first estimates of thermoregulatory requirements for molting Weddell seals in the current climate regime. Establishing these measures is the first step for future assessments of how the physiology and energy budgets of these animals may be altered in a rapidly changing climate. Such information will be critical for the conservation and management of polar pinnipeds and top predators in the changing Southern Ocean environment.

**Figure 1:**
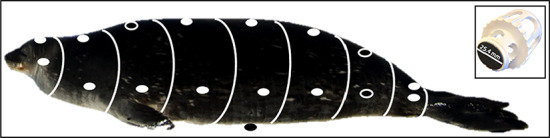
Surface temperature (ST) and heat flux (HF) measurements are shown as circles and were taken at dorsal and lateral points along the seal (from head to tail) at the ears, neck, axillary, sternum, middle, umbilicus, pelvis and ankle sites. Open circles indicate where ST and HF were measured before and after the fur was shaved; the dark circle indicates where a ventral measurement of ST and HF was taken. Inset to the right shows the HF sensor with embedded thermocouple in the custom-made PVC sensor holder. Blubber thickness was measured in the same locations as ST and HF measurements, as were girth, height and width measurements. White rings around the seal delineate the area over which each point measurement was extrapolated for whole body average ST and HF calculations. Figure adapted from Shero *et al.*, (2014).

## Methods

### Experimental set-up

During austral summers 2013–2016, 93 adult female Weddell seals of known age (10–20 years old) and reproductive history from the Erebus Bay, McMurdo Sound population (~S77°68′, E166°82′) were studied. Only female seals were included in this study, as it was part of a larger investigation of life history trade-offs and reproductive physiology in multiparous individuals (Shero, 2015; Beltran *et al.*, 2019). Following established protocols, seals were captured and sedated with a mixture of ketamine and midazolam by veterinary staff based on initial mass estimates of the animal [target induction dose rate of 2 mg/kg ketamine and 0.1 mg/kg midazolam (Mellish *et al.*, 2010)]. This drug combination has a fairly minimal impact on thermoregulatory ability during sedation (Ikeda *et al.*, 2001), and attending veterinarians never noticed any signs that seals were thermally compromised during handling procedures. Initial captures were performed prior to molting onset (November/December, Nov/Dec), and females were relocated and recaptured ~ 60 days later during the molting season (January/February, Jan/Feb). Fieldwork was conducted under the University of Alaska Anchorage’s Institutional Animal Care and Use Committee permits #419971 and 854089 Marine Mammal Protection Act permit #17411, and the Antarctic Conservation Act permit #2014-003.

At each handling, the molt status of the animal was visually assessed and categorized. No animals handled in Nov/Dec were visibly molting, and all were given a ‘pre-molt’ status. Seals handled during the Jan/Feb field season that were not yet visibly molting were given a molt status of 0 (MS0); they were grouped separately from Nov/Dec animals because histological examination has shown that their hair follicles have advanced further in the molt cycle than pre-molt seals (Kirkham in prep). Animals were considered in molt status 1 (MS1) if they had new hair only on the head and face or a thin ‘dorsal stripe’ of new fur along the spine, molt status 2 (MS2) if they had a thick connected stripe of new fur, molt status 3 (MS3) if they had a much wider dorsal stripe of new fur along the shoulders and back that descended onto the flanks and molt status 4 (MS4) if they had no old fur remaining. For further description and images of each molt status see Beltran *et al.*, (2019).

Within ~ 15 min of full sedation, seals were weighed (M, tripod sling suspension, MSI-7300 Dyna-Link 2, ± 0.25 kg resolution) and measured. The seals were marked at eight locations along their length (ears, neck, axillary, sternum, middle, umbilicus, pelvis and ankles) and morphometrics (girth, height, width, distance from nose, all measured ±0.5 cm), blubber thickness (B, measured ±0.5 cm), ST (°C) and HF (W/m^2^) measurements (details below) were taken at each of those locations (Fig. 1). Blubber thickness was measured using imaging ultrasonography (SonoSite Edge ultrasound and C60x/5-2 MHz convex transducer, SonoSite Inc., Bothell, WA). The surface area (SA, m^2^) over which heat could be lost was calculated using measurements of standard and curvilinear length, height, width and girth following the truncated elliptical cones model (Shero *et al.*, 2014; Beltran *et al.*, 2018). Each truncated cone was divided into dorsal/ventral and left/right lateral sides [32.2 and 17.8% respectively, Fig. 1, (Hindle *et al.*, 2015)]. To calculate average HF and ST across the whole body, each HF or ST point measurement (taken on top of the dry-fur) was assumed to be representative of the entire truncated cone section in which the measurement was taken. The whole-body average HF or ST per unit area was then determined by summation, was this value that was used in all further analyses. Lastly, at the time of each handling, ambient temperature (AT, °C), relative humidity (RH, %) and wind speed (WS, m/s) were measured using a Kestrel 4000 Pocket Weather Tracker, which was factory calibrated within 12 months of use. Horizontal solar radiation (SR, W/m^2^) values within 15 min of the handling times were obtained from an Eppley PSP (NOAA) land-based instrument at McMurdo Station (McMurdo Stn, 2018), which is no more than a ~ 15-km distance from where animals were handled.

### Heat flux and skin temperature measurements

Standard heat flux sensors (HFSs) with an imbedded thermocouple (25.4 mm diameter, Concept Engineering, Old Saybrook, CT) were used to measure total HF (mV, MAS830B multimeter, Concept Engineering) and dry ST early in the sedation procedure (directly after weighting the animal). Only seals that had been hauled-out long enough for their fur coat to dry completely were used in this study. The HFS was mounted into a modified PVC holder (Fig. 1) which (i) allowed the sensor to be held against the animal yet remain insulated from the human hand and (ii) allowed for ambient conditions to be maintained on the upper side of the sensor. Only the outer, non-active, rim of the sensor was in contact with the PVC holder. A MM200 temperature gauge (KLEIN tools) was used for field seasons 2013–2015 and HH801 (Omega Engineering) temperature gauge was used for the 2016 field season. Both temperature gauges were calibrated in the lab using a circulating cold water bath (Neslab RTE 7, Thermo Scientific) at 2° increments from 4 to 36°C and paired with a hermetically sealed tip insulated thermocouple (HSTC-TT-T-24S-36-SMPW-CC, Omega Engineering) with accuracy of ±0.4% reading. The HFS was calibrated with factory settings at Concept Engineering, and all measurements are reported as the converted values from multimeter mV to standard units of W/m^2^. To determine if an additional correction factor was needed for the HF measurements, an *in situ* study was performed using a thermally constant heating plate (HP-150-PL, Auber Instruments) and temperature controller (HP-150, Auber Instruments, accuracy ±0.1°C). Measurements from a single HFS were compared to measurements taken from the upper sensor in a set of two stacked HFS in order to determine the insulative capacity of the sensor itself. HF measurements were taken in ambient air temperatures (21°C) on the heating plate at temperatures ranging from 25 to 37°C in 2° increments for five trials. The single (expected value) and stacked (observed value) sensor results were plotted as a regression, and the slope of the line (1.0 ± 0.1) was treated as the correction multiplier in all subsequent analyses (Willis and Horning, 2005; Hindle *et al*., 2015).

In the field, sensors mounted on their holder were first held flat and lightly pressed on the dry-fur of sedated seals to allow the sensor to reach stabilization (between ~ 120 and 180 s) and subsequently moved between data collection points (Fig. 1) to measure HF and ST (~20 s at each location). Thus, the HFS was dry and equilibrated to ambient and seal conditions at the start of the readings. Following the equilibration period, dry-fur readings were taken at each location shown in Fig. 1, for a total of 16 dry-fur surface measurements per animal. Ventral measurements were only collected in 2016, but were extrapolated for previous years using a multiple regression model that included all intrinsic and extrinsic factors (see below). Direct HF and ST measurements were also taken on 79 females from shaved skin patches in three locations: axillary dorsal, pelvis dorsal and pelvis lateral. The insulative capacity of the fur was then calculated as the difference between each HF and ST measurement prior to and ~ 1 min after shaving of the dry-fur (i.e. ST_insulation_ = ST_fur_ − ST_shaved_), using the direct sensor.

In order to assess changes in the thermoregulatory requirements across the molt, HF from obligatory thermoregulation (HFTR = HF − BMR) was calculated following methods from Hindle *et al.* (2015), where BMR was a standard literature value for basal metabolism for resting Weddell seals in-air [1.35 W/kg, (Williams *et al.*, 2004)], and HF was that determined in this study from the direct HF measurements made on dry-fur. We did not directly measure BMR, but used a fixed value in both seasons, as Weddell seals maintained similar behavioural patterns throughout the study and were not fasting during the molt. We also assumed there was no added heat from locomotion or feeding, as the animals were hauled-out of the water, dry and resting. Evaporative water loss was excluded from analysis, as it is generally low, and we aimed to focus on heat loss from the seal trunk.

### Infrared thermal imaging

To determine differences in heat signatures across all molt statuses without direct contact, infrared thermal images (IRT) of seals were taken with a *FLIR T650* series camera that was factory-calibrated annually. Seals (*n* = 169) resting on ice were photographed prior to any disturbance and included those handled in sedation procedures plus additional free-ranging seals of similar molt statuses and apparent condition resting hauled-out nearby. Environmental data (as described above) was collected when photographs were taken. If hand-held ambient weather data were not available for unhandled seals, data from the nearby (~1 km) land-based weather stations at Scott Base (Weather Underground, 2018) were matched to images, providing data were collected within 30 min of time of imaging. All IRT images were reviewed, and only those in which the seals were in focus, within ~ 3 m of the camera, and lying perpendicular to the angle of the camera lens were retained. Seals also had to be dry and have no ice or snow on their fur. When these criteria were met, the best single full-body visible and in-focus image of each seal was retained for analysis.

**Figure 2:**
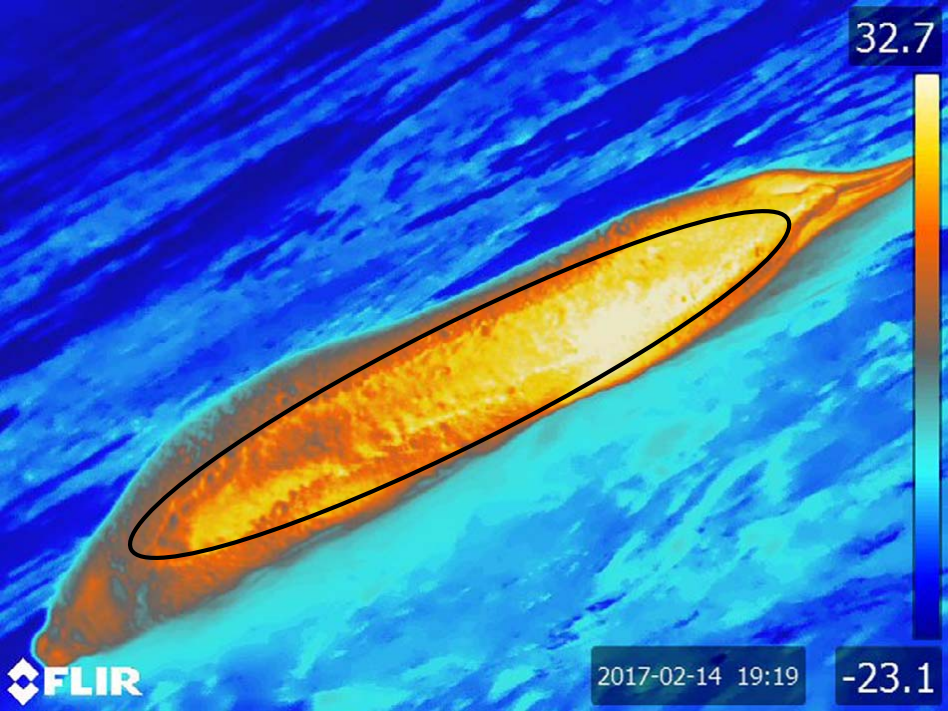
Infrared thermal (IRT) image of a resting seal in molt status 2. The black oval indicates the region of interest (ROI) used to calculate average IRT temperature as described in the methods. Colour indicates ST (°C).

Using *FLIR ResearchIR* software, an oval ‘region of interest’ (ROI) was inscribed around the dorsum of the seal in each selected image (Fig. 2). The head, flippers and outer rim of the seal were omitted from the ROI to avoid the increased heat signatures of the face and to avoid biases due to the decreasing angle of incidence around the edges of the seal (Speakman and Ward, 1998; Nienaber *et al.*, 2010). The average IRT ST calculated inside each ROI was provided by FLIR software statistics package based on pixel coloration and an assumed emissivity for the fur of 0.98, hereafter referred to as IRT. To verify the fur’s emissivity value, a 20 × 20-cm section of seal skin with fur (sculp) was imaged at room temperature (15 repeated trials) at the same time that ST was measured directly by thermocouple. Half of the sculp was spray-painted black matte of a known emissivity (0.95), and half was left unpainted. The temperature of each side was assessed using *FLIR ResearchIR* software assuming a 0.95 emissivity for the painted section and a 0.98 emissivity for the unpainted side. IRT sculp temperatures were additionally compared to direct thermocouple measurements to assess accuracy of IRT against direct thermocouple measurements.

**Table 1:** Number of animals in each molt status for each data set: all handled seals (H), handled seals that were infrared imaged (IRT), handled seals that had shaved patches (SP) and unhandled (UH) animals used in only IRT analysis.

**Season**	**Molt status**	**# (H, IRT, SP, UH)**	**Mass (M, kg)**	**Surface area (SA, m** ^**2**^ **)**	**Blubber thickness (B, cm)**
Nov/Dec	Pre-molt	93, 89, 47, 0	336.1 ± 8.6	3.49 ± 0.05	4.11 ± 0.10
Jan/Feb	0	27, 29, 10, 13	295.7 ± 6.4	3.25 ± 0.04	3.24 ± 0.10
Jan/Feb	1	25, 25, 12, 19	339.8 ± 9.3	3.55 ± 0.07	3.68 ± 0.14
Jan/Feb	2	9, 8, 2, 21	395.1 ± 22.0	3.94 ± 0.17	4.29 ± 0.26
Jan/Feb	3	8, 7, 1, 13	385.1 ± 14.8	3.68 ± 0.13	4.58 ± 0.22
Jan/Feb	4	11, 11, 7, 21	363.1 ± 13.6	3.70 ± 0.12	4.16 ± 0.14
	**Total**	**173, 169, 79, 87**	**336.7 ± 5.4**	**3.50 ± 0.03**	**3.94 ± 0.07**

A given handled seal could be included in H, IRT and SP counts. Average mass (M, kg), surface area (SA, m^2^) and blubber thickness (B, cm) ± standard error are presented for each molt status for all handled individuals.

### Statistical analyses

Sedation dosages were compared (mg/kg) with a non-paired *t* test within and across seasons to insure uniformity in administration. To determine how HF and ST varied in response to intrinsic variables (M, SA, B, MS) and extrinsic variables (AT, WS, RH, SR), a linear mixed-effects (LME) model approach was used. Prior to modelling, variables were first assessed for normality using Shapiro–Wilks tests and collinearity using regressions, which indicated all variables were independent. Animal identification number was included in models as a random effect since individuals were handled twice. Molt status was maintained as an ordered factor using six levels (one for each molt status). Whole body ST, HF and IRT averages were compared across ambient conditions using simple linear regressions and were also compared between molt statuses using Bonferroni pairwise comparisons to test how average values varied as molt progressed. All average values are presented as ± standard error. *R*^2^ decomposition was also performed to assess the relative importance of each factor. Top predictive models of ST, HF and IRT were ranked based on Akaike information criterion (AIC) scores, where all possible models were generated involving each of the intrinsic and extrinsic factors mentioned above. A full list of the models is provided in supplementary materials. Further, IRT surface temperatures in a subset of the handled seals were modelled to identify the factors that most influenced IRT results. Heat flux from thermoregulation (HFTR) was calculated for each molt status and compared between molting and non-molting phases with an unpaired *t* test. All statistical analyses were performed in *R* [version 3.2.2, (R Core Team, 2017)]; *R*^2^ decomposition used package *relaimpo*, LME models used package *lme4* and Bonferroni pairwise comparisons used package *multcomp*.

## Results

### Animal and environmental parameters

Of the 93 seals handled initially in Nov/Dec, 80 were successfully re-handled in the Jan/Feb field season (Table 1). Animals were in various stages of the molt progression and ranged in size from 211–554 kg, averaging 336.7 ± 5.4 kg. They had an average blubber thickness of 3.9 ± 0.1 cm and surface area of 3.5 ± 0.1 m^2^ (Table 1). Analysis of changes in body condition and mass across the molt were beyond the scope of the study, but mean values are provided for informative purposes. Photos from 89/93 females handled in Nov/Dec, 80/80 handled in Jan/Feb and 87/169 free-ranging/un-handled (UH) seals met the criteria for IRT analysis. Initial sedation dosages averaged 2.04 ± 0.01 and 0.10 ± 0.001 mg/kg for ketamine and midazolam respectively. There were no significant differences in dose rates (ketamine: *F*_181.61_ = −0.008, *P* > 0.9; midazolam: *F*_179.15_ = 0.673, *P* > 0.5) within or across seasons, suggesting that seasonal trends in ST and/or HF are not attributable to differential responses to sedation.

**Figure 3:**
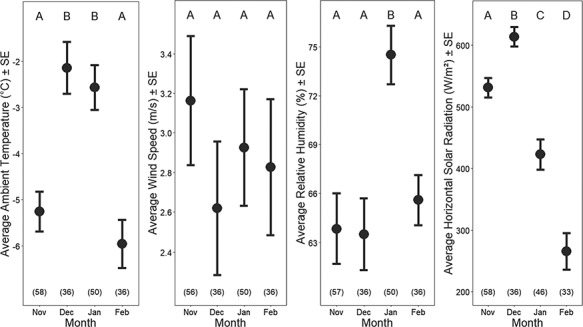
Average (± SE) environmental conditions measured during animal handling in each study month: (**a**) ambient temperature (AT, °C), (**b**) wind speed (WS, m/s), (**c**) relative humidity (RH, %) and (**d**) horizontal solar radiation (SR, W/m^2^). Animal handling was only conducted during the day under mild ambient conditions, so these values do not reflect the average conditions for the month. Significant differences between months are shown with different letters at the top of the panel, and sample sizes for each month are shown in parentheses at the bottom.

As expected, weather conditions during animal handlings varied seasonally. Ambient temperatures (*F*_3,163_ = 13.78, *P* < <  0.001) and horizontal solar radiation levels (*F*_3,163_ = 41.27, *P* < <  0.001) were higher during December and January as compared to November and February (Fig. 3). Relative humidity was highest in January (*F*_3,163_ = 7.131, *P* < 0.001), but wind speed remained relatively consistent throughout the study period, averaging 2.9 ± 0.2 m/s. Although the reported environmental conditions were summarized for only the periods in which animals were handled, trends appeared to match those of the overall weather dynamics in the area on a monthly basis (SCAR, 2018).

### Surface temperatures

**Figure 4:**
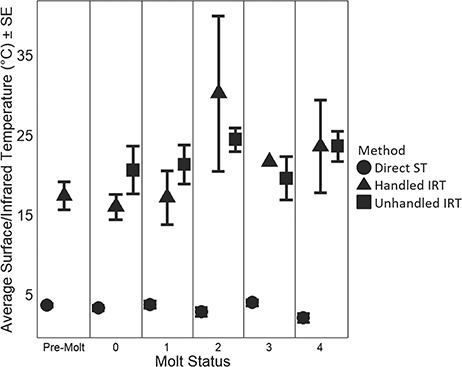
Average ± SE directly measured dry-fur dorsal STs (°C) (circles) and infrared imaged dorsal STs (°C) for handled and unhandled animals (triangle and square respectively). There was no difference in ST measured in animals that were not handled as compared to those that were imaged by IRT prior to handling, however there were significant differences between IRT and direct ST measurements.

Direct ST measurements taken on dry-fur indicated that, on average, seal STs remained a relatively constant 11.2 ± 0.3°C warmer than the environment, independent of season or molt status. Thus, while ST averaged 7.1 ± 0.3°C (range −1.9 to 21.6°C), it was higher on warmer and less windy days, with these two factors accounting for relatively similar amounts of the attributed variance [44.6 and 41.7% respectively, (Table 2)]. Increasing RH and SR were associated with increased ST, but were weaker predictors than AT and WS. Blubber thickness was also retained in top models, and animals with thicker blubber layers had warmer STs. Unexpectedly, MS was not retained as a significant factor in top models, and seals did not maintain higher ST or ∆T during molt (Fig. 4). On average, fur-covered STs were 1.4 ± 4.1°C warmer than those in shaved patches. However, old fur provided slightly (1.1 ± 0.4°C) less insulation than new fur regardless of molt status, as indicated by comparing the STs of new fur covered skin vs old fur-covered skin with direct measurements (Welch two-sample *t* test, *t*_84.75_ = −2.01, *P* value < 0.05). Furthermore, the shaved measurements did not indicate any immediate vasoconstriction on the skin of the seals, as there was no consistent change in the sign of the voltage readings before stabilization had occurred.

**Table 2:** The top three linear mixed-effect models as ranked by AICc scores, and the first model after a ∆AICc > 2 was achieved.

Variable	Predictor variables	LogLik	AICc	∆AICc	Weight	*R* ^2^
ST	**AT** WS **RH SR HF** --- ---- **B**	- 375.909	770.9	0	0.177	0.80
	**AT** WS **RH SR HF** --- **SA** --- ---	−376.437	772.0	1.06	0.104	0.81
	**AT** WS **RH SR** --- --- **SA** --- ---	−377.701	772.3	1.35	0.090	0.81
	**AT** WS **RH SR HF M** ---- **B**	−375.904	773.2	2.25	0.057	0.80
						
IRT-handled	**AT** WS ---- SR ---- --- ---- B MS **dHF** ----	−134.392	298.3	0	0.349	0.69
	**AT** WS **RH** SR ---- **M** ---- --- MS **dHF** ----	−133.601	299.8	1.46	0.168	0.52
	**AT** WS ---- SR ---- **M** ---- --- MS **dHF** ----	−135.195	299.9	1.60	0.156	0.61
	---- WS ---- SR ---- **M** ---- B MS **dHF dST**	−134.685	301.9	3.63	0.056	0.50
						
IRT-unhandled	---- WS ---- **SR** MS	−596.061	1213.5	0	0.516	0.43
	---- WS RH **SR** MS	−595.844	1215.4	1.86	0.203	0.43
	**AT** WS ---- **SR** MS	−595.857	1215.4	1.88	0.200	0.46
	**AT** WS RH **SR** MS	−595.660	1217.3	3.82	0.076	0.44
						
HF	AT ---- RH ---- **ST** --- --- --- MS	−869.152	1761.9	0	0.055	0.28
	AT ---- RH SR **ST** --- --- --- MS	−868.224	1762.4	0.46	0.043	0.30
	AT ---- RH ---- **ST** M --- B MS	−867.060	1762.5	0.48	0.043	0.30
	AT ---- RH ---- **ST** --- **SA** --- MS	−869.119	1764.2	2.25	0.018	0.28

Models for direct-contact ST (°C), IRT determined ST (IRT, °C) and HF (W/m^2^) are shown. Predictors include the extrinsic factors of ambient temperature (AT, °C), wind speed (WS, m/s), relative humidity (RH, %) horizontal solar radiation (SR, W/m^2^), and the intrinsic factors of molt status (MS), mass (M, kg), surface area (SA, m^2^), average blubber thickness (B, cm) and ST (°C) or HF (W/m^2^). Models used dorsal ST and HF (dST and dHF) in the IRT models of handled animals, which were based on images of animals taken prior to sedation. Models of unhandled IRT were limited to extrinsic factors and molt status, as those animals were not handled later for full physiological measurements. Animal ID was retained as a random effect in all models. Factors whose increase led to an increase in the variable of interest are shown in bold, whereas factors whose decrease led to an increase in the variable of interest are unbolded. All *P* values were less than 0.0001.

### Infrared imaging

Tests to determine the appropriate emissivity value to use for IRT calculations revealed that there was no statistical difference between IRT temperatures measured for normal (‘pre-molt’ *ɛ* = 0.98) and painted fur (*ɛ* = 0.95). However, the emissivity of the dull-brown old hair present during molt in Jan/Feb was not measured. If this worn fur had a lower emissivity, IRT estimates of ST in molting seals could be upwardly biased by as much as 4.2%. However, dorsal temperatures calculated within the ROI were an average of 15.1 ± 1.4°C warmer than temperatures measured directly with the thermocouple (Fig. 4, Welch paired *t* test, *t*_68_ = −8.12, *P* < <  0.0001). This difference is far greater than could be accounted for by discrepancies in emissivity due to fur condition alone. Linear mixed-effect models revealed that IRT temperatures were significantly influenced by AT, WS, SR, HF, B and MS (*P* < 0.001). The retention of MS within the model was surprisingly different from the model generated for direct ST measurements and suggests IR and direct contact sensors are measuring two different aspects of the animal’s ST. There were no thermal windows present on the trunks of hauled-out seals visible in the IRT images.

### Heat flux

**Figure 5:**
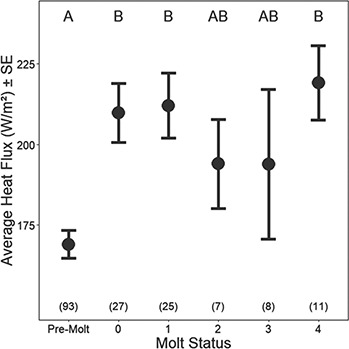
Average HF (W/m^2^) ± SE values as measured on dry-fur across the molt progression of handled seals. Significant differences between each molt status are shown with different letters at the top of the panel, and sample sizes for each molt status are shown in parentheses at the bottom.

While overall HF measured on dry-fur averaged 186.2 ± 3.8 W/m^2^, it ranged from a low of 86.0 to a high of 357.2 W/m^2^. HF was strongly influenced by both intrinsic and extrinsic factors (Table 2). Molt status explained the largest amount (66.5%) of variance in HF (*F*_5,167_ = 7.12, *P* < <  0.001, Fig. 5), and there was a 25.0% increase in HF from pre-molt to early molting stages. Ambient temperature accounted for an additional 23.8% of the variance, with molting seals losing an additional 2.9 W/m^2^ per degree decrease in AT. Solar radiation and WS had no effect, and RH accounted for 5.6% of the variance in the top model. Other than molt status, no intrinsic conditions (M, B, SA) were retained in the top model, but ST accounted for 4.0% of variance. The difference between dry-fur HF and _shaved_HF was on average 111.3 ± 132.8 W/m^2^, which suggests that fur reduces HF by 33%. Furthermore, there was a difference in HF measured on old fur and new fur-covered sites, with old fur providing less (48.3 W/m^2^) insulation than new fur (Welch two sample *t* test, *t*_85.08_ = −2.66, *P* value < 0.01). The obligatory energetic costs of thermoregulation (HFTR) averaged 41.5 ± 5.1 W/m^2^ (0.45 ± 0.05 W/kg) for those animals handled in the pre-molt period. The HFTR doubled during the active molting period, averaging 81.0 ± 5.7 W [0.86 ± 0.06 W/kg, (Welch two sample *t* test, *t*_148.98_ = −5.49, *P* value << 0.001)], but did not differ among active molting categories. If one assumes that the measures of HF are a good approximation of resting metabolic rate (i.e. RMR = HFTR + BMR), then the HFTR accounts for 25% of RMR during pre-molt, and 38% of RMR during the molting period (RMR = 1.80 ± 0.06 vs 2.20 ± 0.06 W/kg).

## Discussion

This study provides baseline measurements of the thermoregulatory costs associated with molting in the most-southerly living mammal, the Weddell seal (Stirling, 1969). Overall, results indicate that despite their suite of physiological adaptations to the polar environment (Young, 1976; Stirling, 1977), Weddell seals experience increased thermoregulatory costs during the molt, and these costs increase during colder, windier conditions. Our findings therefore support the idea that ambient conditions can provide selective pressure for the phenology of molt and indicate that the already high cost of molt may be further exacerbated by environmental changes. Seals that molt later in the season experience rapidly decreasing ambient temperatures and heightened wind speeds (Valkonen *et al.*, 2008) and therefore could lose more heat to the environment than those that molt earlier in the summer. Seals that spend more time in the water likely also have higher energetic costs because the higher conductivity of the liquid media will further increase HF rates (Bowen, 1926; Nadel, 1984). Changing conditions within the Southern Ocean may require that seals spend more time foraging (Siniff *et al.*, 2008; Costa *et al.*, 2010) to regain mass lost during the reproductive period (Ortiz *et al.*, 1984; Stewart and Lavigne, 1984). The associated reduction in time spent hauled-out could delay or extend the molt into late summer/early fall when weather conditions are much colder, thus increasing energetic costs. This suggests that Weddell seals molt so soon after the reproductive period to take advantage of the relatively benign ambient conditions that exist during the short summer season, minimizing HFTR.

### Surface temperature and infrared imaging

While ST was not directly linked to molt status, seals maintained a relatively constant temperature gradient with ambient conditions, even while molting and later in the season when AT decreased and WS increased. This finding echoes prior studies and suggests that polar species maintain relatively cool STs in order to prevent high rates of heat loss (Irving and Krog, 1955; Barnes, 1989; Prestrud, 1991; Blix, 2016). Furthermore, ST remained near operative temperature [a predictive ST incorporating AT, WS and SR (Campbell and Norman, 1998)] throughout the molting season, even as environmental conditions became colder. However, the average shaved patch skin temperature of 8.5 ± 4.11°C was far below the optimal temperature for epidermal mitosis (35°C) that Feltz and Fay (1966) determined for a variety of temperate and polar pinniped species. While they observed mitosis at temperatures as low as 17°C in harbour seal (*Phoca vitulina*), bearded seal (*Erignathus barbatus*) and Steller sea lion (*Eumetopias jubatus*) cultured skin cells, no cells showed mitotic activity at 4°C. However, Weddell seal dry-fur STs were frequently far cooler (range: −1.9 to +21.6°C; median during visible molt: 6.5°C), suggesting that the Weddell seal molt can progress at much lower temperatures than in other species. Still, since cell division and hair growth likely occur more rapidly in warmer skin, molting when AT is high (and when sunlight can further warm the skin) would be advantageous (McCafferty *et al.*, 2005; Norris *et al.*, 2010). The energetic savings accrued by molting during the long, warm, sunny days shortly after the breeding period may therefore be a main driver in the molt phenology of Weddell seals.

The surprising finding that IRT estimates of ST were significantly greater than temperatures determined by direct measurement, and much larger than could be accounted for by errors in assumed emissivity, strongly suggests that the two methods measured fundamentally different things. While the direct measurements captured skin ST, the IRT images captured the fur ST, which was likely much warmer than the seal’s skin due to SR warming the fur itself. Direct ST measurements do not include this warm air layer because the thermocouple is depressed onto the animal’s surface during measurement. Additionally, the measuring device itself blocks incoming radiation for the duration of the stabilization and measurement period (Fig. 1). In contrast, much of the long wave SR that hits a seal’s fur coat can be reradiated back off the animal as infrared (Dawson *et al.*, 2014; Dawson and Maloney, 2017) and may be captured in an IRT image. Thus, the higher apparent IRT STs are the only measurement to include heat from both trapped air and long wave reradiated infrared radiation.

Solar radiation interference and air trapped in fur also likely explain why molt status was a significant factor in IRT models: if the dark new fur was heated more by short-wave radiation (and thus reradiated long wave radiation) than old fur or was more effective at trapping air, animals with a larger amount of new fur would have higher IRT readings for a given ST than those in earlier or pre-molt statuses. This is supported by studies which have found differences in fur structure and quality to reflect differing amounts of solar reflectivity (Walsberg, 1988; Walsberg and Schmidt, 1989). Despite these potential biases, IRT may still be useful for the detection of thermal windows or ‘hot spots’ (Mauck *et al.*, 2003; Erdsack *et al.*, 2012; Paterson *et al.*, 2012), provided images are not taken of seals in direct sunlight. However, we did not detect any thermal windows along the trunk by IRT (nor were there large regional differences in ST measured by direct contact), which suggests that basking Weddell seals are not overheating or releasing large amounts of heat to the environment.

### Heat flux and thermoregulatory costs

HF was primarily impacted by molt status, and was higher in molting seals as compared to seals in pre-molt. This was especially evident in earlier molt statuses, as HF increased by 26.4% from pre-molt to molting status 0 to 2. Changes in HF occurred even as ST remained relatively consistent, which is only possible if there were also changes in the conductivity of the skin (Ogle, 1970). Because blood is a much better heat conductor than dry skin (Love, 1980; Grimnes and Martinsen, 2015), the most likely way for this to occur is through changes in dermal perfusion. While we were unable to measure dermal perfusion, our observations of increased HF at a given ST in molting seals is consistent with a molt-specific increase in blood flow to active hair follicles. It was not surprising to find that the highest HF measurements were taken on seals in early molt statuses, as this is the time when hair follicles are in anagen phases and hair fibres are being actively synthesized (Kirkham in prep)]. The associated increase in dermal perfusion results in a decrease in the insulative capacity of the skin itself. In addition to molt status, environmental conditions also influenced HF, with higher rates on colder, more humid days which most often occur later in the season. The effect of AT and RH was evident despite the fact that HF measurements were only collected during relatively mild conditions when animals could be handled safely. Because weather conditions deteriorate rapidly in the late summer/early fall period (NOAA, 2018), seals that initiate molt later in the season almost certainly have higher rates of heat loss as compared to the handled seals. The effects of ambient conditions on molt costs, independent of seal ST, may be a key selective pressure on molt timing.

Regardless of the underlying cause, the increase in HF rates in molting seals resulted in a more than 2-fold increase in average HFTR. This HFTR has the potential to increase the RMR during the Weddell seal molt by up to 22%, provided there is no alternative offsetting change in BMR. Increases in RMR of up to 41% during molt have been observed in Northern fur seals [*Callorhinus ursinus* (Hansen *et al*., 1995; Donohue *et al*., 2000)]. That the molting costs for Weddell seals were slightly smaller is not surprising given their shorter hair type and more rapid molt. We suggest that the most likely causes for the increased RMR during molt are the change in heat conservation adaptations. Increased skin perfusion and a reduced blubber layer during molt likely increase the lower critical temperature of the thermal neutral zone. A significant change in the lower critical limit could drive an increase in the RMR for individuals in cold polar climates. In addition, increased tissue synthesis costs above that of basal tissue maintenance would increase metabolic costs as well. Reductions in RMR have been documented in species during periods of significantly reduced activity (long lactation or molting haul-out periods) [ex. elephant seals (Costa *et al.*, 1986; Slip *et al.*, 1992), harbour seals (Ashwell-Erickson *et al.*, 1986) and grey seals (Anderson and Fedak, 1985)]. These long haul-out periods when animals are fasting drive reductions in BMR (Keys *et al.*, 1950; Guppy *et al.*, 1994) as a mechanism to reduce energy demand, thereby conserving protein and lipid stores (Castellini and Rea, 1992). However, Weddell seals continue to forage regularly during molt and do not dramatically change their behaviour, so therefore a change in the metabolic strategy and reduction in RMR is unlikely.

We acknowledge that the HFTR (metabolic difference between RMR and BMR) may include other costs such as cost of activity, digestion and evaporative water loss. However, those alternate costs are likely low because the animals handled were dry (not recently feeding), inactive (resting on the surface of the ice) and likely not experiencing significant respiratory water loss (breathing regularly through their nose, with moisture recaptured via nasal turbinates). Furthermore, each of these additional costs is not expected to differ seasonally and therefore would not contribute differentially to early season versus late season HF. Similarly, any potential biases introduced by sensor design or sedation would be similar across seasons. In contrast, HFTR was influenced by ambient conditions, which suggests it is comprised of mostly those costs which are also influenced by ambient conditions (i.e. thermoregulation and not feeding or activity). The cost of HFTR was highest in early molt statuses, suggesting that seals which begin to molt later in the season likely experience much higher thermoregulatory costs as compared to seals that molt earlier in warmer conditions.

While phocids typically haul-out for longer durations during the molt, Weddell seals do not significantly reduce foraging time, but instead spend a considerable amount of time in the water foraging throughout the mid-summer and molting periods (Harcourt *et al.*, 2000; Burns and Kooyman, 2001; McIntyre *et al.*, 2013). Because thermal conductivity of water is 25 times that of air (Schmidt-Nielsen, 1984; Dejours *et al.*, 1987), diving activity is likely associated with increased heat loss (Willis & Horning 2005; Hindle *et al.*, 2015) relative to that when hauled-out. However, foraging during mid-summer and throughout the molt may be necessary so that seals can recover the large amount of mass lost during the breeding season (Wheatley *et al.*, 2008; Beltran, 2018). Foraging success rates during the summer period are much higher than during winter months likely because prey are shallower and productivity higher (Cherel *et al.*, 1997; Plötz *et al.*, 2002; Burns *et al.*, 2008). Seals that forage extensively during the mid-summer when prey are most accessible may be able to reduce the amount of time they spend in the water, thereby minimizing periods of very high heat loss without significant reductions in energy intake rates.

Despite the high cost of molting and the associated loss of foraging opportunities while hauled-out, all pinnipeds molt annually (Beltran *et al*., 2018). While the fur of adult phocids does not provide much insulation in-water (Frisch *et al.*, 1974), it does provide 29–34% of the total thermal resistance in-air for harp and hooded seals respectively (Kvadsheim and Aarseth, 2002; Pearson *et al.*, 2019). Similarly, our direct measurements of the difference in HF between furred and shaved spots indicate that in adult Weddell seals, fur provides 32% of total thermal resistance in-air, with new fur providing slightly more insulation than older fur. The replacement of all damaged fur in a single short window allows Weddell seals to avoid higher thermoregulatory costs that would accompany a later, or more prolonged molt period. This optimization of molt timing allows for seals to minimize excess energy expenditures for growing a new, more insulative, fur coat.

### Molting in a changing climate

In the context of the overall energy budgets for Weddell seals, molting is a significant energetic cost. This study has contributed to our understanding of the costs of the annual pelage molt to provide insights into how environmental factors influence thermoregulatory requirements. Over the next century, climate patterns in the Southern Ocean are expected to change significantly, and the magnitude and direction of these changes vary both regionally and seasonally (Turner and Overland, 2009; Gardner *et al.*, 2018). Impacts on Southern Ocean pinnipeds will be mediated by climate directly, as well as by changes in sea ice (as haul-out substrate) and prey availability (Simmonds and Isaac, 2007; Constable *et al.*, 2014; Kelman, 2015). While Weddell seals exhibit a circumpolar distribution and remain close to the continent in fast-ice regions (Stirling, 1971), other pinnipeds such as leopard (*Hydrurga leptonyx*), crabeater (*Lobodon carcinophagus*) and Ross (*Ommatophoca rossii*) seals rely more heavily on pack-ice surrounding the continent as haul-out substrate for molting. Therefore, these Southern Ocean pinniped populations, which likely have similar molting thermoregulatory requirements, may respond to climatic shifts in way that reflect regional differences in sea ice patterns. For example, in Eastern Antarctica, the extent and duration of the summer sea ice is projected to increase (Turner *et al.*, 2009; Comiso *et al.*, 2011; Stammerjohn *et al.*, 2012), which could benefit molting seals by providing them a stable haul-out substrate. In contrast, Western Antarctic seasonal ice cover has been substantially reduced in recent years and is expected to keep declining (Bingham *et al.*, 2012; Pritchard *et al.*, 2012). Reduced sea ice cover could increase the amount of time seals spend in the water and change haul-out and foraging locations during the molt (Forcada *et al.*, 2012). While warmer ambient temperatures may reduce thermoregulatory costs, they likely also will have negative impacts on ice extent and prey availability (Ducklow *et al.*, 2007) for Southern Ocean pinnipeds.

Most work on potential impacts of climate change and environmental variability to Weddell seals has focused on the reproductive period [and the ability to successfully support offspring (Hadley *et al.*, 2007; Proffitt *et al.*, 2007; Beltran *et al.*, 2017)]. This study has demonstrated that molting is also a time of high energetic demand. Changes to local environment that (i) increase heat loss, (ii) alter ability to haul-out during molt or (iii) reduce ability to regain blubber between reproduction and molt will all negatively impact seals by increasing energy costs. Similarly, anything that causes delayed molt onset or a longer molt period (such as might occur if seals need to reach a mass/condition threshold before molting) will also increase costs. This could occur in a regime of reduced Southern Ocean productivity (Constable *et al.*, 2014; Kelman, 2015) if seals were forced to spend more time foraging to build sufficient mass reserves to complete the energetically expensive molt. In addition, later molts are associated with lower probability of becoming or retaining pregnancy (Beltran *et al*., 2019), so additional carryover consequences exist as well for poorly timed molting. As a result of regional variation in climate trajectories and associated ecological effects, it is likely that different populations of Weddell seals will experience different types of stressors due to the effects of climate change. For example, East Antarctic populations of Weddell seals may experience greater environmental stressors leading to higher metabolic costs due to colder environmental conditions, whereas populations of West Antarctic Weddell seals may experience more biological stressors of reduced prey availability. While polar pinnipeds will face different challenges, understanding the baseline summertime energetic requirements for molting is critical for understanding how these animals will adapt or decline in a changing climate.

## Supplementary Material

Supplementary_coaa022
